# Bisucaberin B, a Linear Hydroxamate Class Siderophore from the Marine Bacterium *Tenacibaculum mesophilum*

**DOI:** 10.3390/molecules18043917

**Published:** 2013-04-02

**Authors:** Masaki J. Fujita, Koji Nakano, Ryuichi Sakai

**Affiliations:** 1 Creative Research Institution, Hokkaido University, Hakodate 041-8611, Japan; 2 Graduate School of Fisheries Sciences, Hokkaido University, Hakodate 041-8611, Japan

**Keywords:** siderophore, *Tenacibaculum mesophilum*, macrocycle, bisucaberin

## Abstract

A siderophore, named bisucaberin B, was isolated from *Tenacibaculum mesophilum* bacteria separated from a marine sponge collected in the Republic of Palau. Using spectroscopic and chemical methods, the structure of bisucaberin B (**1**) was clearly determined to be a linear dimeric hydroxamate class siderophore. Although compound **1** is an open form of the known macrocyclic dimer bisucaberin (**2**), and was previously described as a bacterial degradation product of desferrioxamine B (**4**), the present report is the first description of the *de novo* biosynthesis of **1**. To the best of our knowledge, compound **1** is the first chemically characterized siderophore isolated from a bacterium belonging to the phylum Bacteroidetes.

## 1. Introduction

Siderophores are bacterial products that have affinity for ferric ion and are responsible for iron uptake and transport [1]. Iron is an essential element for almost all organisms, serving as a cofactor for various enzymes. However, the bioavailability of ferric ion in the marine environment is low and thus it is a limiting factor in bioproduction. In addition to acting as ionophores in iron-deficient environments, siderophores also play important roles in bacterial chemical communication. In the marine environment, some bacteria acquire siderophores produced by the other strains for their own growth [2] in a process known as siderophore piracy [3]. It is hypothesized that under the pressure of intense competition for iron, bacteria have evolved various siderophore biosynthesis and utilization machineries to overcome siderophore piracy or to enable use of siderophores for specific inter–strain chemical communication [4,5].

**Figure 1:**
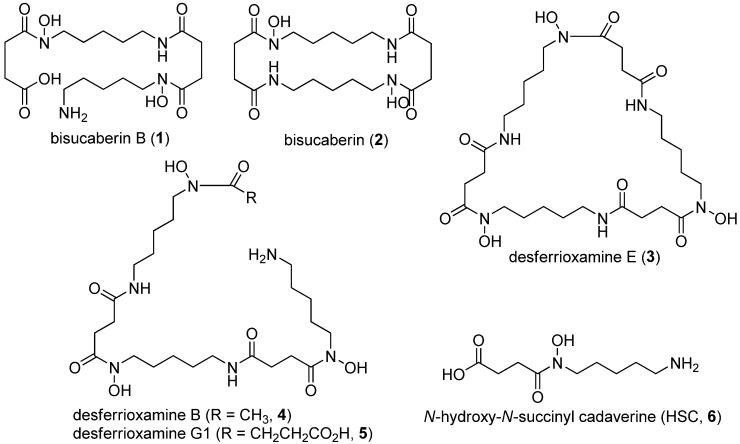
Macrocyclic and acyclic *N*-hydroxy-*N*-succinyl cadaverine (HSC) based siderophores and their common monomer HSC.

Siderophores also serve as model compounds in biosynthetic research. For example, studies of the enterobactin from *Escherichia coli* and vibriobactin from *Vibrio cholera* have contributed to understanding of non–ribosomal peptide synthase (NRPS) [6]. Recently, biosynthetic enzymes of siderophores such as bisucaberin (2) [7] and desferrioxamines (**3**–**5**) [8] which contain *N*-hydroxy-*N*-succinyl cadaverine (HSC, 6) as a general constituent (HSC-based siderophores) were described as a new class of amide bond-forming enzymes [9]. The HSC-based siderophores are a series of compounds macrocyclic or acyclic HSC oligomers with or without some modifications (Figure 1). To date, several biosynthetic genes for HSC-based siderophores have been cloned from various bacteria and the marine metagenome. Heterologous production of bisucaberin (**2**) through expression of a cloned gene cluster using *E. coli* as the host was also reported [10]. However, the mechanisms for regulating oligomerization and macrocyclization reactions have yet to be elucidated.

Recently, we isolated a bacterium, *Tenacibaculum mesophilum*, which produces the linear HSC-based siderophore bisucaberin B (**1**). Although compound **1** was identified as an *Azospirillum irakense*-mediated degradation product of desferrioxamine B (**4**), no detailed characterization of the molecule was reported [11]. In the present study, we found **1** as a single siderophore product from the bacterium. Interestingly, known HSC-based siderophores are mostly macrocyclic or linear with acetyl or other monocarboxylate capping on the hydroxylamino-terminal preventing macrocyclization; thus, exclusive production of linear **1** possessing a free carboxylic acid is unusual. Here, we report the isolation of this bacterium, describe the analysis, purification, and elucidation of the structure of compound **1**, and discuss its biosynthesis and related chemical ecology perspectives.

## 2. Results and Discussion

A siderophore-producing bacterium was isolated from an unidentified marine sponge collected in the Republic of Palau. Based upon 16S rRNA sequencing, the bacterium was identified as *Tenacibaculum mesophilum*, belonging to the phylum Bacteroidetes [12]. *Tenacibaculum mesophilum* was first reported as a marine sponge-associated bacterium from both Japanese and Palauan specimens, but a chemical characterization of the organism, including its siderophore production, has not been reported. 

**Figure 2:**
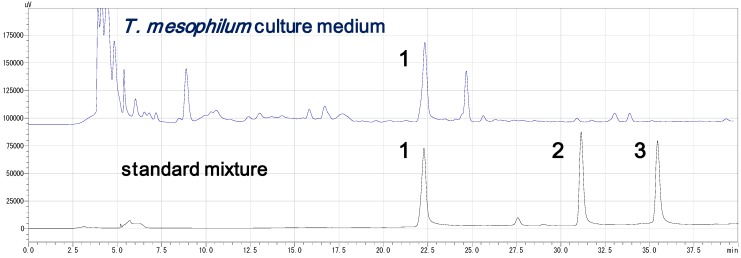
Reversed-phase HPLC chromatograms of *T. mesophilum* culture medium (upper) and a mixture of compounds **1**–**3** (lower). Centrifuged cultured medium was injected directly onto the column without any prior fractionation or concentration steps. The identity of bisucaberin B (**1**) in both samples was confirmed by co-injection. Fractions were collected every 2 min. The siderophore activity was found only in the fraction containing peak 1 in the upper chromatogram.

The bacterium was cultured for four days, after which siderophores were extracted from the medium using C18 gel, and then material bound to the gel was eluted with MeOH. The concentrated extract was fractionated using C18 reversed-phase open column chromatography with stepwise elution with aqueous MeOH. Siderophore containing fractions eluted with 10–50% aqueous MeOH were combined and further separated by size exclusion chromatography on Sephadex G-10 resin eluted with water. The recovered fractions showing siderophore activity were then subjected to HPLC separation, yielding bisucaberin B (1) as a pure material; no other active molecules were detected from the culture medium (Figure 2).

**Figure 3:**
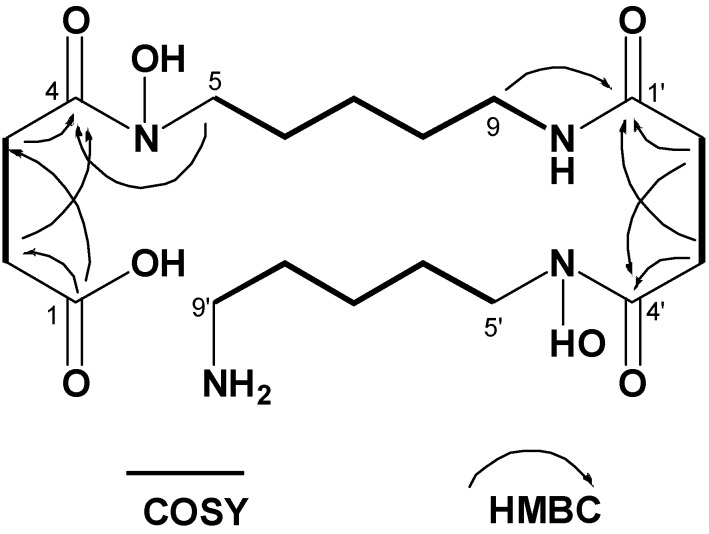
COSY and key HMBC correlations for bisucaberin B (**1**).

Based on HR-ESIMS data, the molecular formula of compound **1** was deduced to be C_18_H_34_N_4_O_7_, which was one H_2_O unit larger than that of bisucaberin (**2**), a macrocyclic HSC-based siderophore originally isolated from the deep-sea bacterium *Alteromonas haloplanktis* [13] and later from *Vibrio salmonicida* [14]. The characteristic proton NMR spectrum of compound **1** consisted of multiple methylene signals and only one exchangeable proton signal, suggesting that it belongs to the HSC-based siderophores (Table 1). To date, several bacterial macrocyclic HSC-based siderophores have been described (Figure 1), most of which have symmetrical repeating di- or trimeric motifs and thus exhibit simple monomer-like 1D NMR spectra. In contrast, all 18 resolved signals were observed in the ^13^C-NMR spectra of compound **1**, suggesting it has an asymmetrical structure. Analysis of the 2D NMR data, including COSY, HMQC, and HMBC, revealed the presence of two *N*-hydroxy-*N*-succinyl cadaverine (HSC) units in the structure, and on the basis of key HMBC correlations from δ_H_ 2.99 (H_2_-9) and δ_H_ 2.29 (H_2_-2') to δ_C_ 171.5 (C-1'), it was determined that the two HSC monomers are connected by an amide bond. The gross structure of compound **1** is elucidated as shown in Figure 3, *i.e*., a ring-opened form of bisucaberin (**2**).

**Table 1:** . ^1^H- and ^13^C-NMR data for compound 1 in CD_3_OD: DMSO-*d*_6_ = 1:1.

	δ_H_ mult. (*J* (Hz))	δ_C_		δ_H_ mult. (*J* (Hz))	δ_C_
1		175.8	1'		171.5
2	2.44 t (6.4)	28.0	2'	2.29 t (6.4)	27.8
3	2.31 t (6.4)	33.0	3'	2.59 t (6.4)	30.3
4		171.9	4'		171.9
5	3.45 t (6.4)	46.1	5'	3.48 t (6.4)	46.6
6	1.47^a^	25.8	6'	1.51 ^a^	25.5
7	1.21 quint. (7.2)	23.1	7'	1.27 quint. (6.8)	22.7
8	1.35 quint. (6.8)	28.6	8'	1.49 ^a^	27.2
9	2.99 quint. (6.4)	38.1	9'	2.71 t (6.4)	38.6
N*H*	7.75 t (6.4)				

a: coupling constants were not determined due to signals overlapping.

The validity of the proposed structure was also supported by positive ninhydrin test results, which indicated the presence of a primary amine. In addition, treatment with trimethylsilyldiazomethane resulted in a 14-mass unit shift in the molecular weight of compound **1**, indicating the formation of a methyl ester. Taken together, these results indicated that compound **1** is a seco-acid of bisucaberin (**2**).

**Figure 4:**
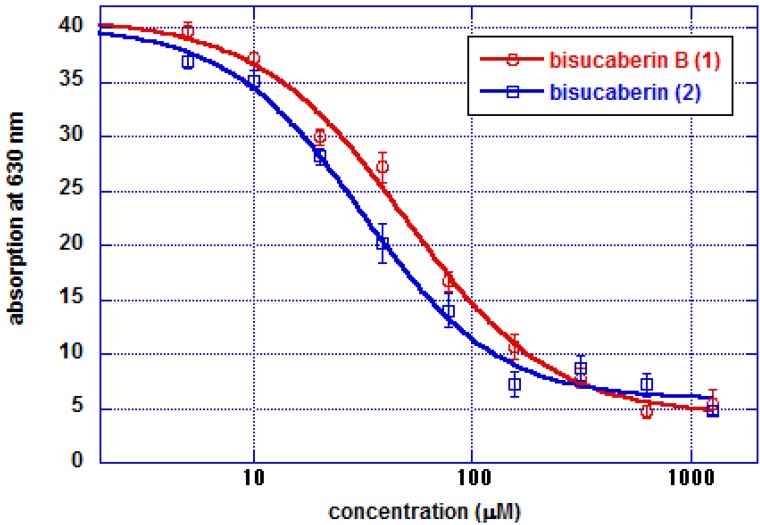
Chrome Azurol S (CAS) assay results. Absorption at 630 nm indicative of the concentration of CAS-ferric ion complex declines due to competition for ferric iron by other chelators. Each data point represents the means ± SD (n = 6).

The ferric ion chelating activity of compound **1** was compared with that of compound **2** using a Chrome Azurol S (CAS) assay [15]. A dilution series of test samples (final concentration 5–1,250 μM) was mixed with CAS assay solution, after which the optical absorption at 630 nm was measured. Both compounds **1** and **2** produced a concentration –dependent attenuation of 630 nm absorption (Figure 4), with IC_50_ values of 55 and 37 μM, respectively, indicating that the chelating activity of bisucaberin B (**1**) is comparable to that of compound **2**.

Bisucaberin B (**1**) was previously reported as a minor metabolite of the bacterial decomposition of desferrioxamine B (**4**) [11]. In the present study, we found for the first time that bisucaberin B (**1**) is biosynthesized *de novo* as the sole siderophore. To date, the trimeric analogue, desferrioxamine G1 (**5**), was reported from several bacteria belong to the genus *Streptomyces* [16], *Pseudomonas* [17], *Erwinia* [18], and *Vibrio* [19]. However, in most cases, compound 5 was co-produced with macrocyclic counterpart **3**, and is thought to be a biosynthetic intermediate of compound **3** [9]. The fact that no trace of a cyclic product was found in the culture medium in our study strongly suggests that compound **1** is formed as a linear final product, and is not the hydrolysate of a cyclic product (Figure 2). 

**Figure 5:**
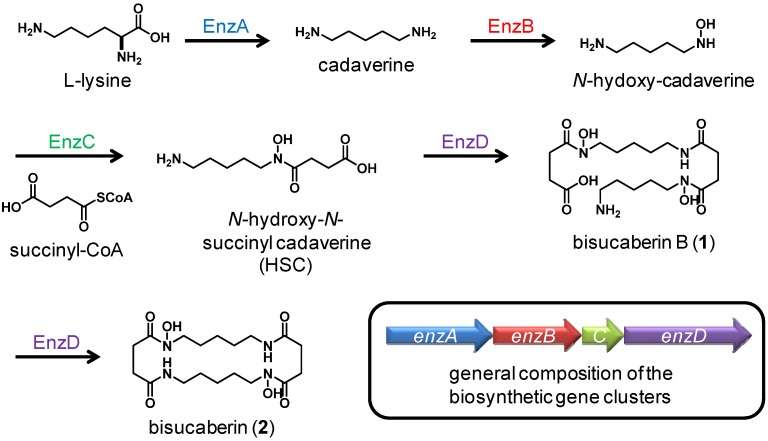
General scheme of HSC-based siderophores biosynthesis and composition of the biosynthetic gene clusters. The fourth enzyme in the *T. mesophilum* is believed to catalyze dimer formation but not macrocycle formation, a previously unreported catalytic feature.

The general biosynthetic scheme and a composition of the biosynthetic gene clusters for HSC-based siderophores are depicted in Figure 5 [9]. The first three enzymes, EnzA-C, catalyze monomer formation, while the final enzyme, EnzD, catalyzes both the oligomerization and macrocyclization reactions. The fact that *T. mesophilum* produces only a linear dimer and no cyclic products suggests that enzyme D of this strain can catalyzes the formation of an amide bond between the monomers and does not catalyze macrocyclization, which if true would represent a previously unreported phenomenon. We expect that a comparison of enzyme D to other known macrocyclization enzymes will help reveal the molecular mechanism of macrocyclization reactions. An in-depth understanding of oligomerization-macrocyclization mechanisms could enable the production of engineered HSC-based siderophores of desired size and structure. 

The ferric ion chelating activity of the linear dimer was comparable to that of its macrocyclic counterpart. We hypothesize that production of the acyclic chelator is a countermeasure to prevent siderophore piracy by other bacteria. Because the conformation of the siderophore-ferric ion complex for compounds **1** and **2** are expected to be different, iron–complexed compound **1** may not be recognized by receptors for compound **2** expressed by neighboring bacteria capable of pirating exogenous siderophores. Thus, a comparison of the selectivity of siderophore receptors for linear and cyclic HSC-based siderophores would be interesting from a chemical ecology point of view. Cloning of the genes encoding the biosynthetic enzymes and putative receptor for compound **1** is in progress.

## 3. Experimental

### 3.1. General Procedures

Low and high resolution ESI mass spectra were measured on a Exactive mass spectrometer (Thermo Fisher Scientific, Waltham, MA, USA). MALDI-TOFMS was measured on a 4700 Proteomics Analyzer (Applied Biosystems, Foster City, CA, USA) using gentisic acid as a matrix. NMR spectra were recorded on a ECP-400 NMR spectrometer (JEOL, Tokyo, Japan) at 400 MHz for ^1^H and 100 MHz for ^13^C in CD_3_OD and DMSO-*d*_6_ (1:1) as a solvent. Chemical shifts of ^1^H and ^13^C-NMR spectra were referenced to the solvent peaks: δ_H_ 3.30 and δ_C_ 49.0 for CD_3_OD. UV spectrum of compound **1** (H_2_O: MeOH = 1:1) and UV absorption of CAS solution assay were measured on an ND-1000 spectrophotometer (Thermo Fisher Scientific). Preparative and analytical HPLC was performed using Prominence HPLC system equipped with photodiode array detector (Shimadzu, Kyoto, Japan). DNA sequence was determined using BigDye terminator cycle sequencing kit (Applied Biosystems) on 3130*xl* Genetic Analyzer (Applied Biosystems). All chemicals were purchased from Wako Pure Chemical Industries (Osaka, Japan) except for those specifically mentioned.

### 3.2. Bacterial Strain

Siderophore producing bacterium, *Tenacibaculum mesophilum* was isolated from an unidentified marine sponge which was collected by hand using SCUBA in the Republic of Palau in March 2012. Sponge specimen was spiked by sterilized bamboo sticks, then spread on sea water-based agar plates (5.0 g yeast extract, 10.0 g tryptone, 10.0 g agar in 1.0 L of sea water), and kept at the ambient temperature for one week. A yellow colored bacterial colony was transferred to a new agar plate as a single strain. Genomic DNA was prepared using a standard method and partial DNA fragment of 16S ribosomal RNA (rRNA) was amplified by polymerase chain reaction using universal primers, 8F (5'-AGAGTTTGATCCTGGCTCAG-3') and 1492R (5'-GGTTACCTTGTTACGACTT-3') [20] to afford 1,444 bp fragment. DNA sequence of the amplified fragment was determined by standard method and homology analysis was performed using Basic Local Alignment Search Tool (NCBI) to 100% match with that of *Tenacibaculum mesophilum* NBRC16307 (accession number, AB681058), originally isolated from unidentified marine sponges collected at Japan and the Republic of Palau [12]. Isolated strain was transferred to the new plates once a month, and kept at room temperature until use.

### 3.3. Culture and Isolation

Siderophore producing strain was pre–cultured overnight in the sea water based medium (1.0 g D-galactose, 6.0 g tryptone, 1.0 g glycerol, 1.5 g yeast extract in 1.0 L of sea water) [21], and then inoculated into the same medium (400 mL in one liter flask), shaken at 30 °C for 4 days at 200 rpm. The cultured medium was centrifuged and passed through C18 resin (Cosmosil 75C18-PREP, Nacalai Tesque, Kyoto, Japan) to extract active molecules, then eluted with MeOH. Obtained active extract was fractionated by C18 gel (same with above) open column chromatography with stepwise gradient elution of pure water, 10% MeOH, 30% MeOH, 50% MeOH, 70% MeOH, and 100% MeOH. Active compound was eluted between 10% to 50% aqueous MeOH fractions. They were all combined and further purified by Sephadex G-10 (GE Healthcare, Piscatway, NJ, USA) column chromatography with water. Activity was found in the middle eluted fractions. Combined active fractions was concentrated, and then a part of the material was subjected to the HPLC purification using reversed phase column (Inertsil ODS-3, GL Sciences, Tokyo, Japan) with aqueous MeOH linear gradient system from 10% to 60% within 30 min to afford bisucaberin B (**1**, 21.3 mg, isolated yield 38.9 mg/L) as a white amorphous solid; UV (MeOH:H_2_O = 1:1) No UV absorption maximum above 224 nm; NMR data, see Table 1; HR-ESIMS *m/z* 419.25019 (M+H)^+^(calcd. for C_18_H_3__5_N_4_O_7_, +0.39 ppm).

### 3.4. HPLC Analysis of the Standard Compounds and Cultured Medium (Figure 2)

HPLC analysis of the both standard mixture and culture medium was performed under the following condition, (Inertsil ODS-3 (4.6 × 250 mm), linear gradient from 10% to 70% MeOH from 2 min to 32 min, flow rate 0.6 mL/min, monitored at 220 nm). Centrifuged supernatant of 4 days cultured medium (50 μL) was directly subjected to HPLC analysis. Identity of compound **1** in both samples was confirmed by co-injection. Fractions were collected every 2 min, and siderophore activity of all fractions was tested by CAS plate assay.

### 3.5. Methylation

Trimethylsilyldiazomethane solution (10% in *n*-hexane, 1 μL) was added to a solution of compound **1** in MeOH (1 mg/mL, 50 μL), kept at room temperature for 3 h. The reaction mixture was directly subjected to the MALDI-TOF MS analysis.

### 3.6. CAS Plate Assay

A CAS plate assay was performed basically according to the reported method [15]. A 10% (v/v) of CAS stock solution (0.1 mM FeCl_3_, 1.0 mM HCl, 1.0 mM CAS, 2.0 mM cetyltrimethylammonium bromide) was mixed with 90% (v/v) of autoclave melted LB agar solution (1% agar at final concentration), and then poured into the empty plates and solidified. Test samples were absorbed on the 6 mm diameter paper disks, and were put on the CAS assay plates. They were kept at room temperature overnight, and then diameter of the resulted yellow halo around the paper discs was measured.

### 3.7. CAS Solution Assay

A CAS solution assay was performed basically according to the reported method [15]. Two fold diluted series of compounds **1** and **2** dissolved in H_2_O (final concentration 5–1250 μM) were mixed with same volume of CAS assay solution (0.6 mM cetyltrimethylammonium bromide, 15 μM FeCl_3_, 150 mM Chrome Azurol S, 0.5 M anhydrous piperazine, 0.75 M HCl) and kept at room temperature for 4 h, and then absorption at 630 nm was measured. Experiments were repeated six times for each sample.

## 4. Conclusions

To date, several macrocyclic and acyclic HSC-based siderophores isolated from various bacteria from the phyla Proteobacteria and Actinobacteria have been described. The present study demonstrated bioproduction of the linear HSC based siderophore bisucaberin B (**1**) by *Tenacibaculum mesophilum*, a member of the phylum Bacteroidetes. It is quite rare that exclusive production of a linear HSC based siderophore without monocarboxylate capping on the hydroxylamino-terminal, and as such it is believed that a characterization of the enzymes involved in its biosynthesis will help elucidate the molecular mechanisms of macrocyclization. To the best of our knowledge, this is the first report describing the chemical characterization of a siderophore isolated from bacteria belonging to the phylum Bacteroidetes.

## Supplementary Materials

Supplementary File 1
