# Supplementary material for: Bisucaberin B, a Linear Hydroxamate Class Siderophore from the Marine Bacterium Tenacibaculum mesophilum
Source: Molecules. 2013 Apr 2;18(4):3917–26. doi: 10.3390/molecules18043917 (PMC6270104; doi:10.3390/molecules18043917)

Supporting Information

Figure S1. ^{1}H-NMR spectrum of bisucaberin B (**1**) in CD_3OD + DMSO *d*-6 (1:1).

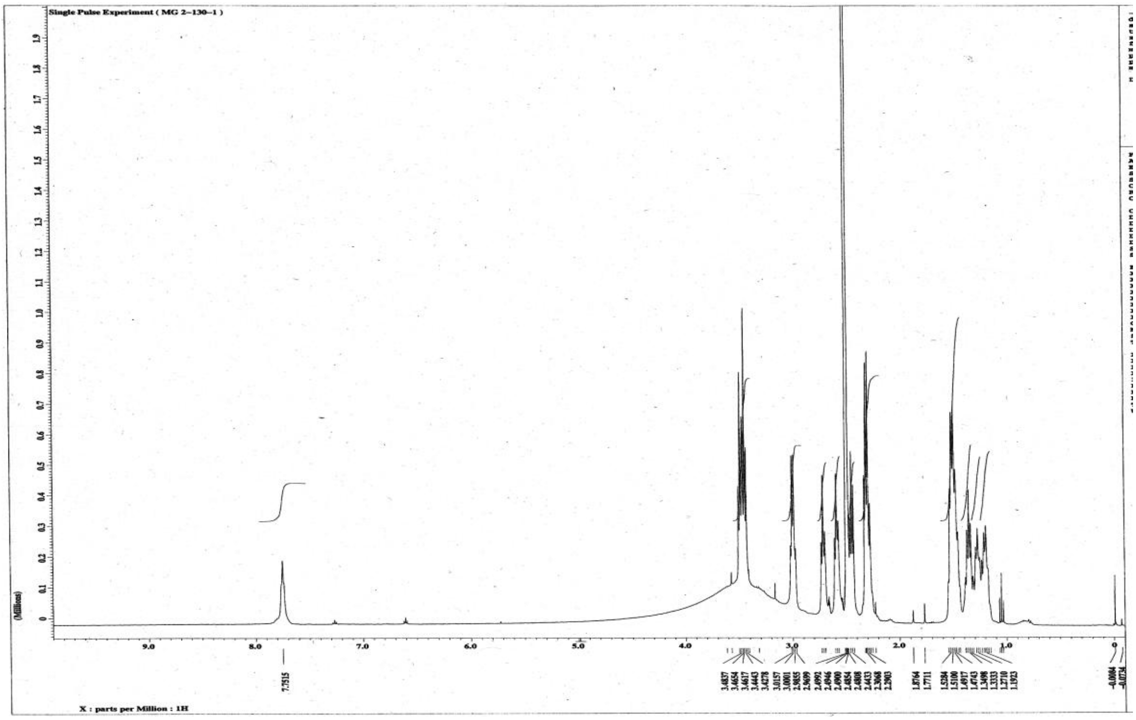

Figure S2. ^{1}H-NMR spectrum of bisucaberin (**2**) in CD_3OD + DMSO *d*-6 (1:1).

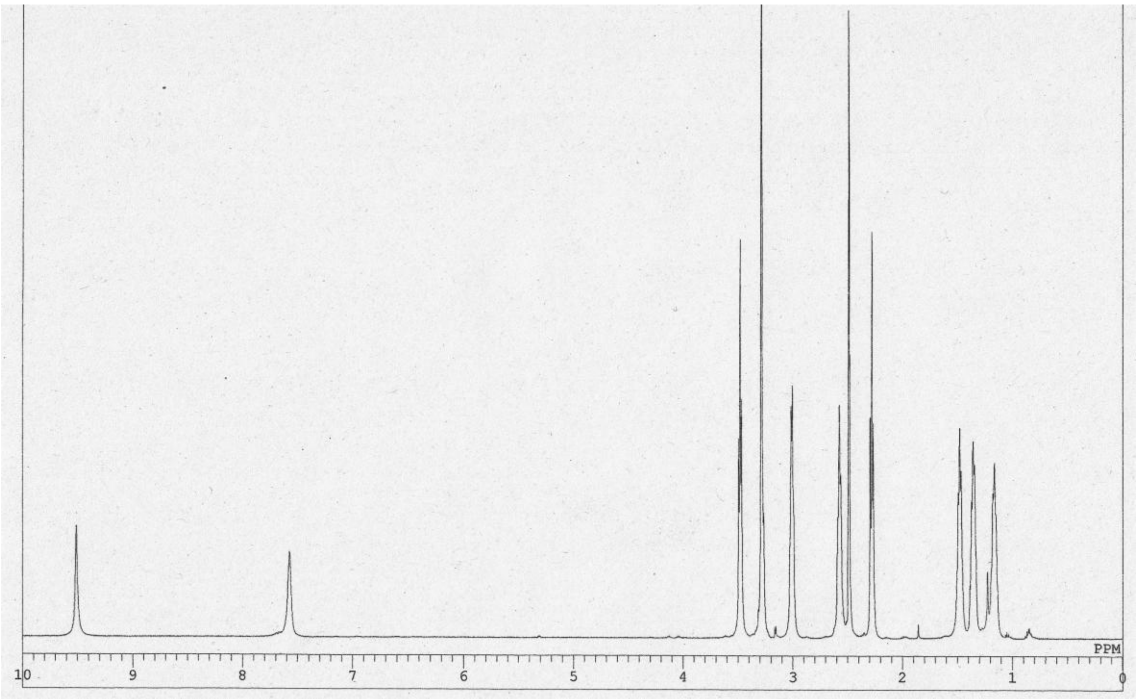

Figure S3. ^{13}C-NMR spectrum of bisucaberin B (**1**) in CD_3OD + DMSO *d*-6 (1:1).

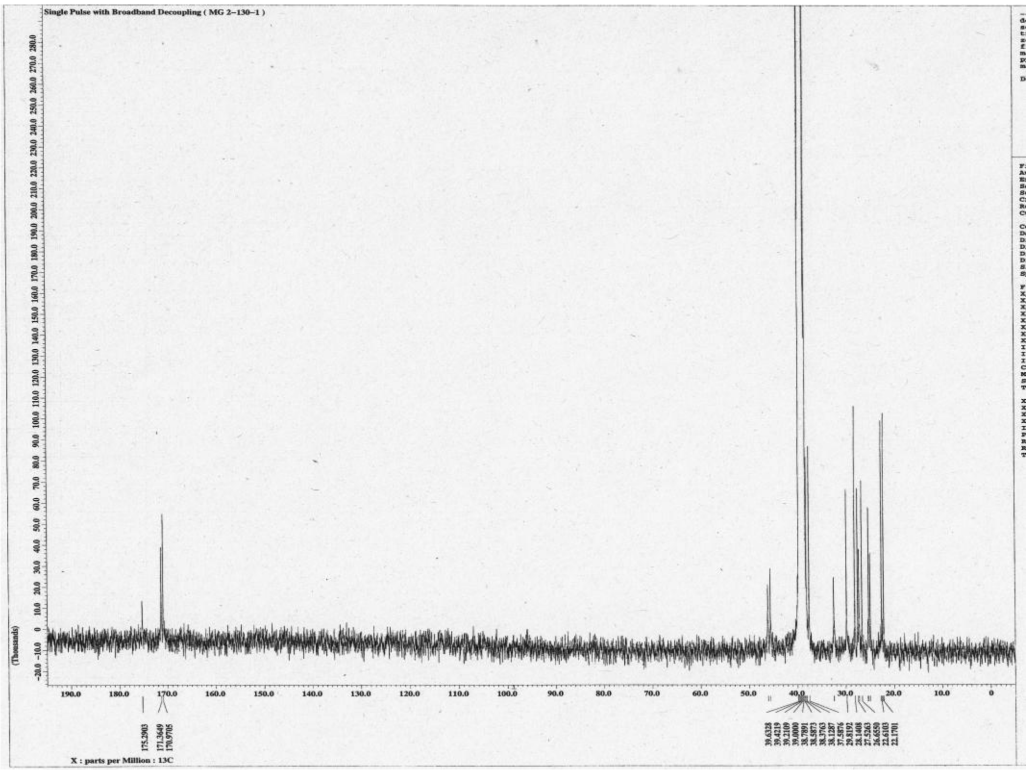

Figure S4. **C**OSY spectrum of bisucaberin B (**1**) in CD_3OD + DMSO *d*-6 (1:1).

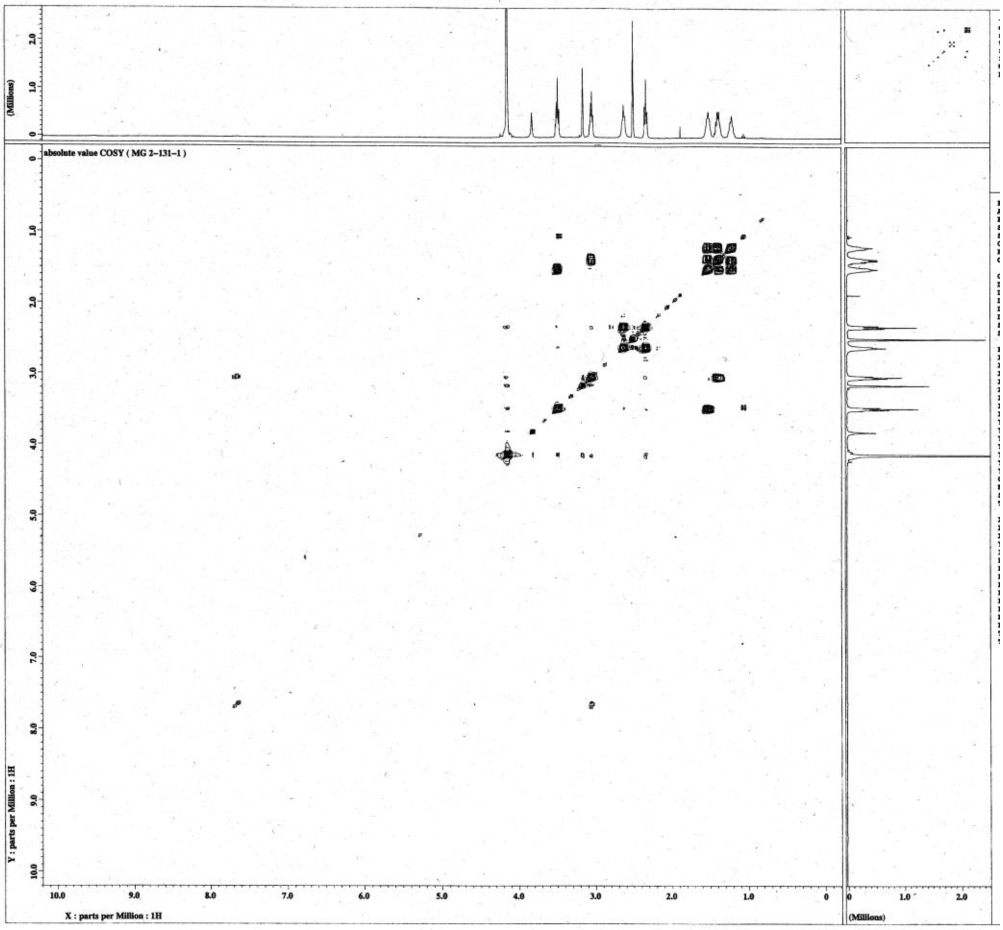

Figure S5. ROESY spectrum of bisucaberin B (**1**) in CD_3OD + DMSO *d*-6 (1:1).

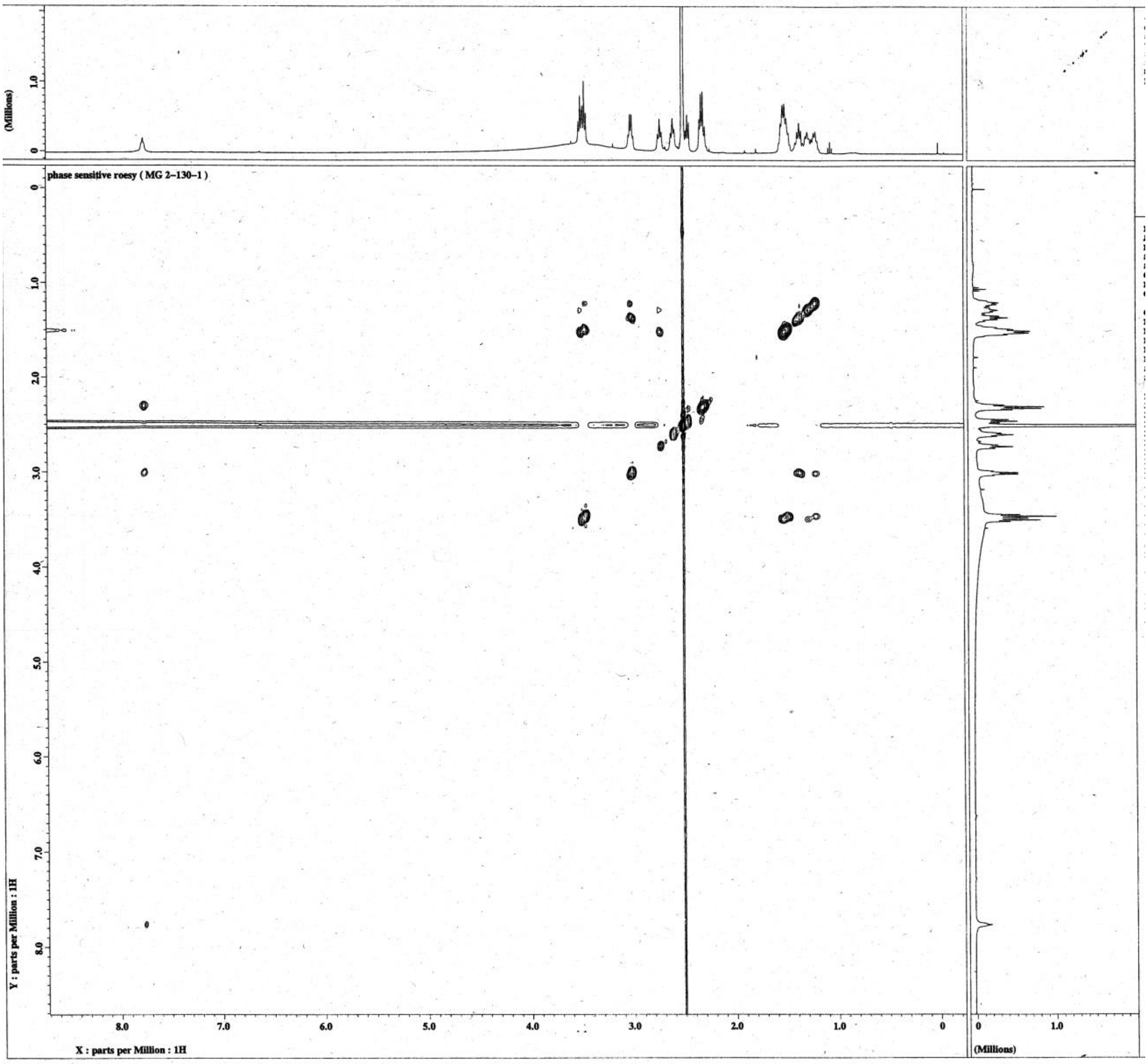

Figure S6. HMQC spectrum of bisucaberin B (**1**) in CD_3OD + DMSO *d*-6 (1:1).

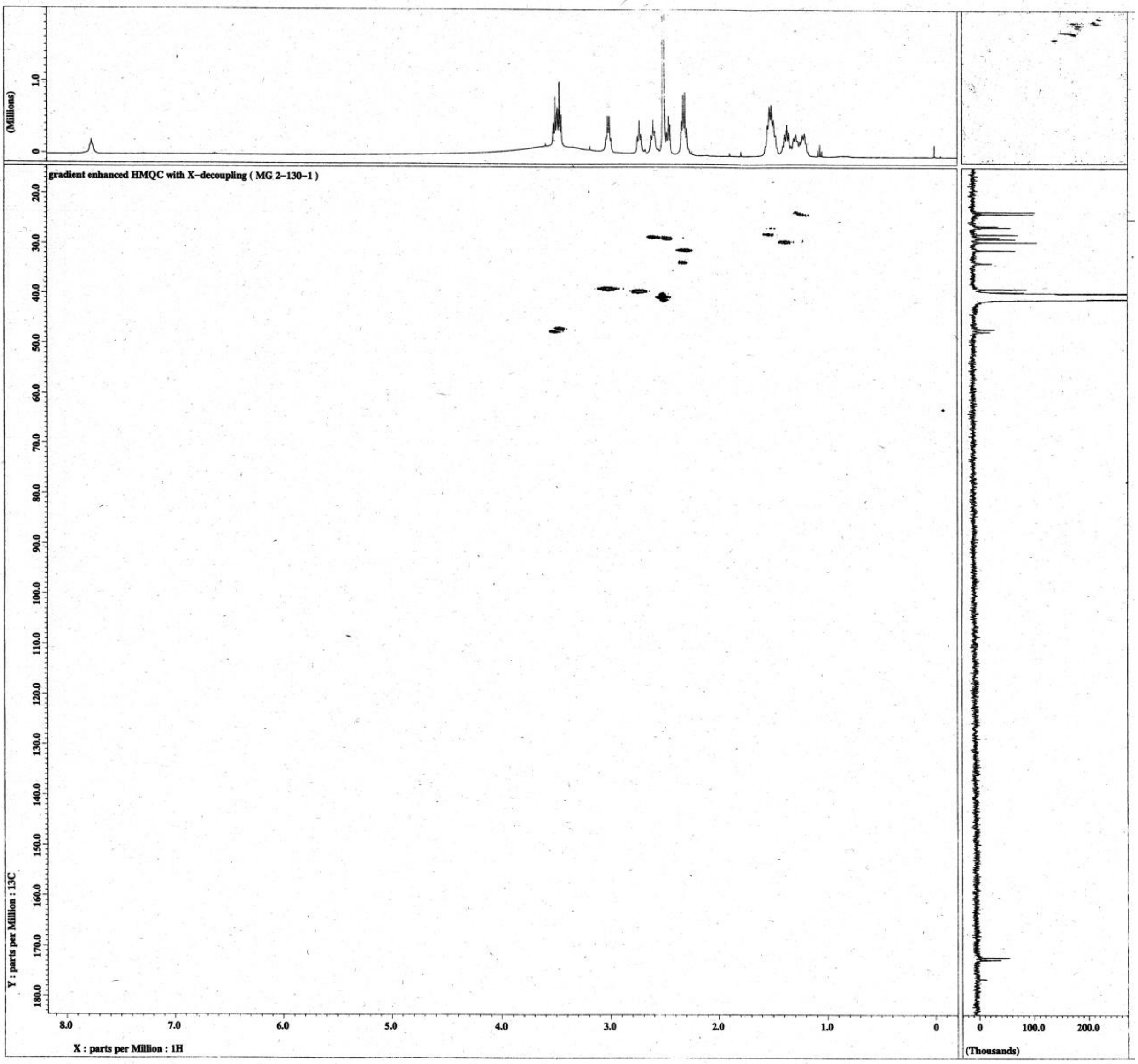

Figure S7. HMBC spectrum of bisucaberin B (**1**) in CD_3OD + DMSO *d*-6 (1:1).

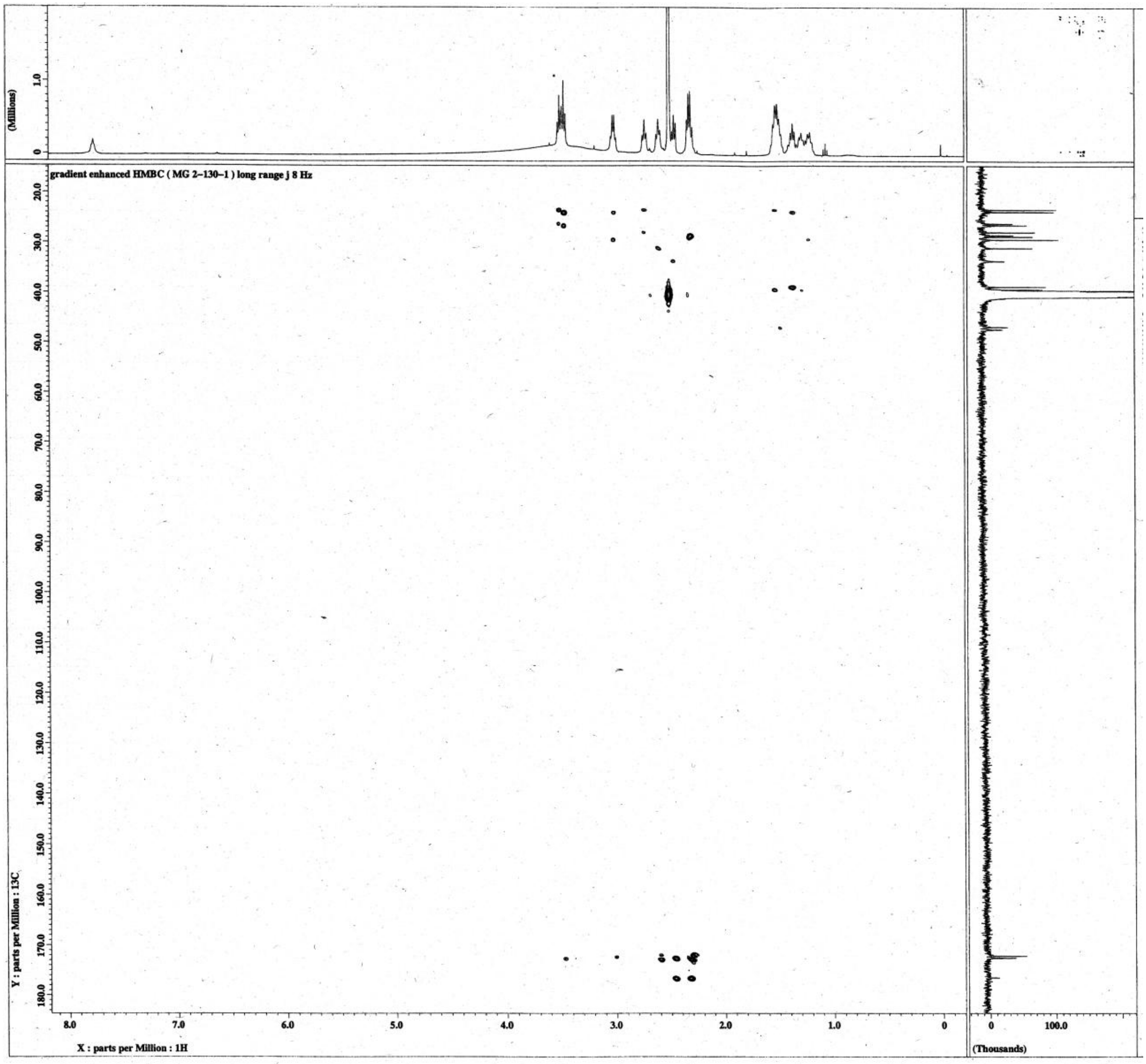

Figure S8. Low-Resolution positive (upper) and negative (lower) mode ESI mass spectra of bisucaberin B (**1**).

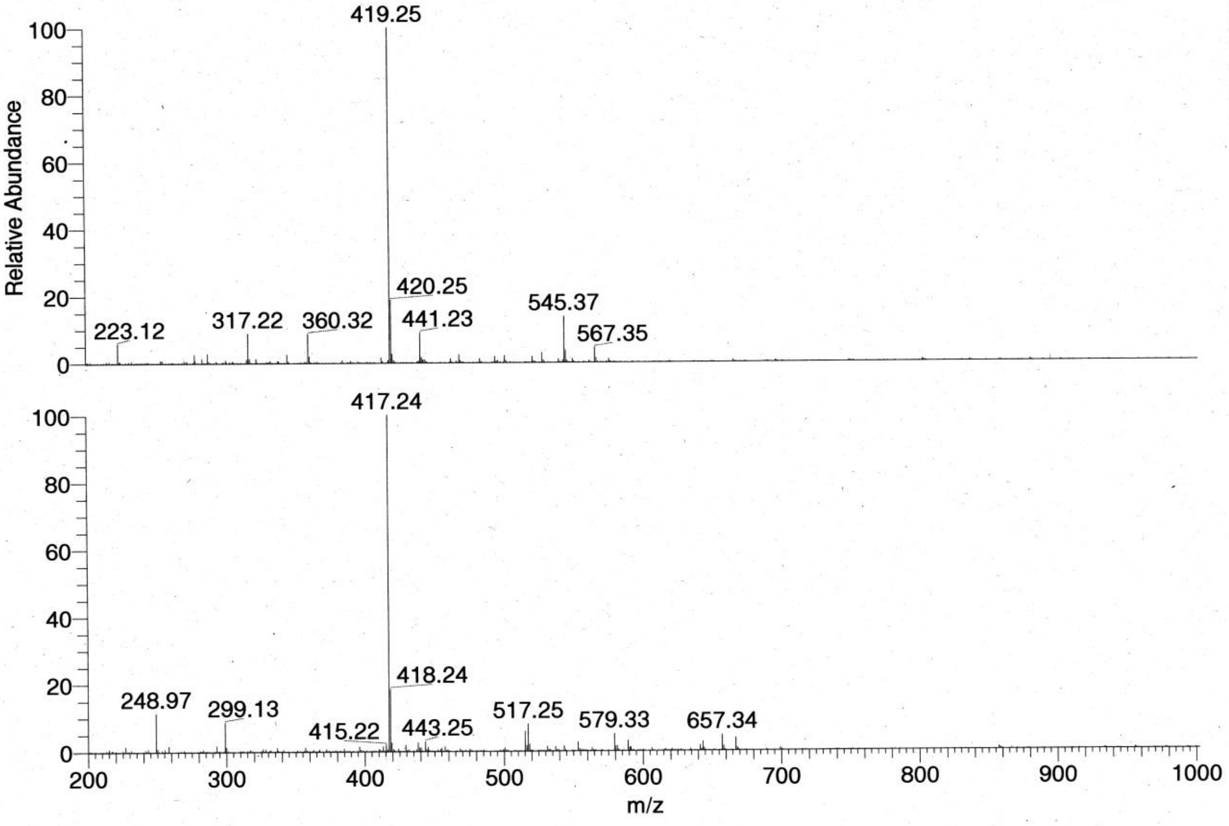

Figure S9. Positive-mode MALDI-TOF mass spectrum of bisucaberin B methylester.

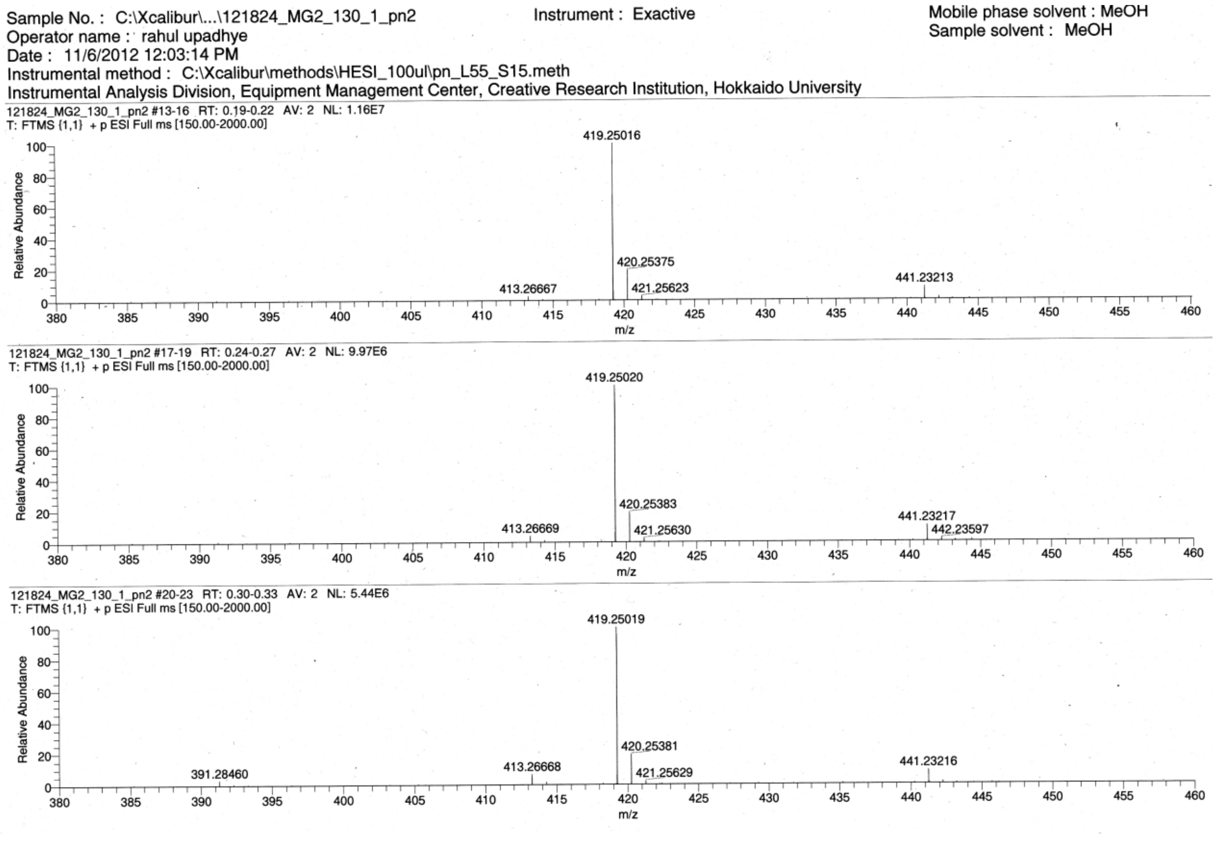

Figure S10. High-Resolution ESI mass spectra of bisucaberin B (**1**).

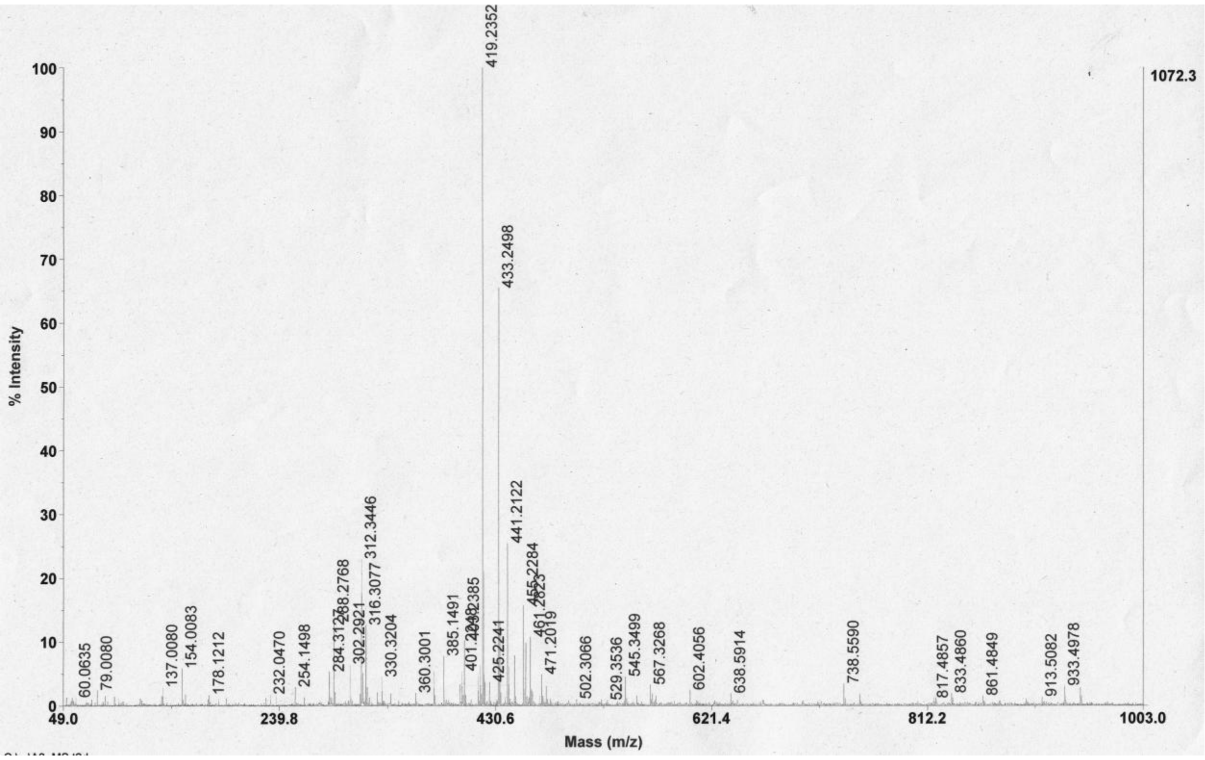

Supplement: Supplementary File 1 [file molecules-18-03917-s001.pdf]
